# Psychosocial hospital work environment and nurses' health assessed with salutogenic indicators: a systematic review

**DOI:** 10.3389/fpubh.2026.1738847

**Published:** 2026-06-16

**Authors:** Aistė Klimanskaitė, Jurgita Andruškienė, Jūratė Grubliauskienė, Marija Truš

**Affiliations:** Faculty of Health Sciences, Klaipė da University, Klaipė da, Lithuania

**Keywords:** nurses, psychosocial work environment, salutogenic health indicators, SHIS, WEMS

## Abstract

**Aim:**

This study aimed to systematically review scientific evidence on how the psychosocial work environment affects nurses' health and well-being through salutogenic health indicators.

**Material and methods:**

The literature search was conducted from February to April, 2025, using the keywords psychosocial work environment, nurses' health, salutogenic health indicators, SHIS, WEMS, nurses' well-being in PubMed and Google Scholar. The search strategy followed PRISMA 2020 guidelines. After applying the exclusion criteria (publication period earlier than 2015 or later than 2024, and publication language other than English or Lithuanian), duplicate removal, abstract screening and quality assessment of the studies, 14 studies published from 2015 to 2024 were included in the final review. The quality of the included studies was assessed using the Joanna Briggs Institute Critical Appraisal Tools, the Mixed Methods Appraisal Tool and the Critical Appraisal Skills Programme. Studies were included if they reached at least 60% of the maximum possible score according to the relevant checklist, corresponding to “moderate to high quality”. Studies that scored below this threshold or did not provide sufficient methodological information were excluded from the final analysis.

**Results:**

Positive psychosocial factors such as social support, autonomy, role clarity, and participatory leadership were consistently linked to improved well-being. For instance, nurses with higher sense of coherence scores reported significantly lower burnout and depression risk, while higher job control and collaboration were associated with better job satisfaction and engagement. Salutogenic Health Indicator Scale scores indicated that up to 60% of nurses experienced frequent physical and emotional symptoms related to workplace stressors, and Work Experience Measurement Scale results highlighted that favorable work environments were strongly associated with higher well-being (*p* < 0.05). In contrast, negative factors, particularly excessive workload, time pressure, lack of managerial support, and workplace conflict were strongly correlated with increased stress symptoms, emotional exhaustion, and reduced motivation across multiple settings.

**Conclusions:**

The psychosocial work environment plays a crucial role in shaping nurses' health and well-being. Strengthening salutogenic resources and addressing adverse psychosocial factors could enhance nurses' resilience and contribute to sustainable workforce retention.

## 1 Introduction

The working environment and professional activities of nurses are closely linked to the quality of healthcare services and the sustainability of the healthcare system. In recent years, there has been a growing emphasis on the importance of the psychosocial work environment of nurses, as this professional group experiences not only a heavy workload but also constant emotional and ethical challenges (1, 2). The global shortage of nurses remains one of the major problems in modern healthcare, and a difficult working environment is one of the factors contributing to staff leaving the profession (3). These trends significantly impact not only on the health and well-being of nursing professionals, but also on patient care outcomes.

The psychosocial work environment encompasses dimensions such as workload, control, social support, sense of justice, and opportunities for professional development. Research shows that unfavorable psychosocial factors are associated with emotional exhaustion, symptoms of stress, risk of depression, and reduced work engagement (4, 5). These factors are particularly important in the healthcare sector, where nurses are constantly confronted with patient suffering, death, and moral dilemmas (2). Systematic reviews indicate that unfavorable psychosocial environment leads to a higher risk of professional burnout, increases staff turnover, and reduces patient safety indicators (6).

At the same time, it is increasingly emphasized that analyzing risk factors alone is insufficient; it is necessary to explore protective and strengthening resources that can help nurses maintain their health in a challenging work environment. In recent decades, health science has increasingly developed a salutogenic approach, which focuses not on the causes of disease but on strengthening health-promoting resources (7, 8). The salutogenesis model is based on concepts such as “sense of coherence,” which encompasses the comprehensibility, manageability, and meaningfulness of life events. A high sense of coherence is associated with better mental health, greater resistance to stress, and more effective coping strategies (9).

Salutogenic factors are particularly important in the working environment of nurses. Recent studies show that personal and organizational resources, such as self-care, professional support, and opportunities to participate in decision-making can significantly reduce psychosocial stress and improve work quality (10, 11). In addition, strengthening nurses' resilience and identifying health-promoting factors are becoming an increasingly important areas of research, as they can help to develop more sustainable workplace interventions (12). A salutogenic approach in healthcare institutions also helps to promote an organizational culture focused on employee well-being and quality of patient care (13).

The global situation of the nursing profession shows that in many countries, nurses experience not only physical but also psychosocial risks, and these risks are often systemic in nature. Studies reveal that working conditions can vary greatly between different healthcare institutions and countries, but common trends—excessive workloads, insufficient staffing, limited opportunities for professional growth, and a lack of organizational justice remain a universal problem (1, 6). These circumstances encourage the search for new ways to not only reduce risks but also strengthen protective health-promoting factors.

Based on these insights, it is necessary to develop research that integrates the analysis of psychosocial risk factors and a salutogenic approach to fully understand the impact of nurses' working environment on their health. Although in recent years more and more studies have examined these aspects in an international context, there is still a lack of sufficient data to better understand the specific factors that help nurses remain healthy and motivated in challenging working conditions (14). Therefore, this study aims to contribute to the expansion of knowledge by analyzing the psychosocial work environment of nurses and the salutogenic factors that influence it.

Aim of research: to analyze the links between the psychosocial work environment in hospitals and nurses' health, assessed using salutogenic health indicators.

## 2 Materials and methods

### 2.1 Search strategy

The study protocol was registered in the PROSPERO database (http://www.crd.york.ac.uk/PROSPERO, ID: CRD42022299665). The systematic literature search was conducted between February and April, 2025. Two electronic databases were used to ensure both high methodological quality and broad coverage of relevant studies: PubMed and Google Scholar PubMed was selected due for its well-developed Medical Subject Headings (MeSH) taxonomy and indexing of peer-reviewed biomedical and nursing research, while Google Scholar was included to capture theses, gray literature, and studies not indexed in PubMed but relevant to the topic of psychosocial work environments.

The keywords and their combinations were developed based on the research question and the PICO framework (15). The following terms were applied: psychosocial work environment, nurses' health, salutogenic health indicators, SHIS, WEMS, nurses' well-being. Boolean operators “AND” and “OR” were used to combine terms and maximize search sensitivity and specificity. Truncations and phrase searches were also applied where appropriate to include variations of the key terms. For example, combinations such as “psychosocial work environment” AND “nurses' health”, or “salutogenic health indicators” OR “sense of coherence” were employed.

The search was limited to publications from 2015 to 2024 to include up-to-date evidence while avoiding outdated methodological approaches. Only studies written in English and Lithuanian were included. Articles published in peer-reviewed journals, dissertations, master theses and high-quality gray literature that met the inclusion criteria were considered eligible.

All records retrieved were imported into Mendeley Reference Manager to facilitate systematic screening and the removal of duplicates. The screening process followed the PRISMA 2020 guidelines (16), ensuring transparency and replicability in systematic reviews. Screening occured in several stages: (1) titles and abstracts were independently reviewed by two researchers against predefined inclusion and exclusion criteria; (2) studies clearly not meeting the research aim, for example, those not related to nursing, not addressing psychosocial work environment, or not using salutogenic indicators were excluded at this stage; (3) full texts of potentially relevant studies were retrieved and evaluated in detail. Discrepancies between reviewers were resolved through discussion and consensus.

The initial search yielded a total of 1,231,152 records (483 from PubMed and 1,230,669 from Google Scholar). After duplicate removal and abstract screening, the vast majority of studies were excluded for irrelevance. Following full-text assessment, only 14 studies met all inclusion criteria and were included in the final review.

Search strings used:

A. PubMed: ((“psychosocial”[All Fields] OR “psychosocial work environment”[All Fields] OR “psychosocial environment”[All Fields] OR psychosocial[All Fields]) AND (nurse^*^[All Fields] OR nursing[All Fields])) AND (salutogenic[All Fields] OR salutogenesis[All Fields] OR “sense of coherence”[All Fields] OR SHIS[All Fields] OR WEMS[All Fields] OR wellbeing[All Fields] OR “well-being”[All Fields] OR “health indicator”[All Fields] OR “health indicators”[All Fields])
B. Google Scholar: “psychosocial work environment” AND (nurse OR nursing) AND (“sense of coherence” OR salutogenic OR SHIS OR WEMS OR wellbeing OR “well-being” OR “health indicator” OR “health indicators”)

### 2.2 Retrieved articles flow

After applying filters for free full text and publication period (2015–2024), 3,728 records remained. Following the removal of duplicates, 3,584 records were screened by title and abstract. At this stage, 3,144 records were excluded as irrelevant, and 440 records were retained for further review.

Out of these, 144 full-text articles were assessed for eligibility based on predefined inclusion and exclusion criteria. During this stage, 61 articles were excluded because of insufficient methodological quality, absence of salutogenic indicators, or lack of relevance to the psychosocial work environment of nurses. As a result, 83 articles were initially considered suitable. A further detailed evaluation revealed that 68 studies did not fully meet the eligibility criteria and were excluded.

**Figure 1:**
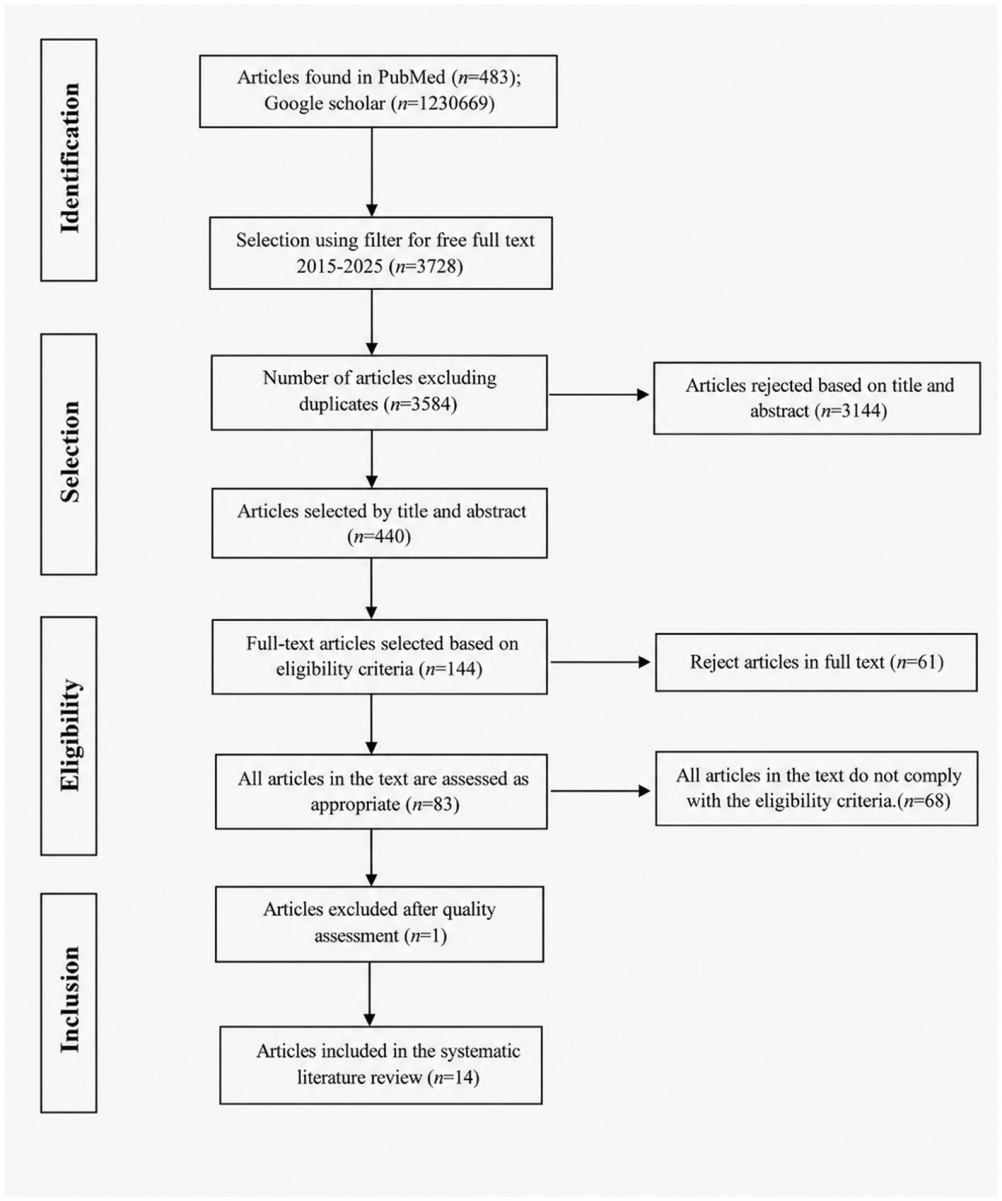
PRISMA 2020 flow diagram illustrating the selection process of included studies.

Finally, 14 studies published between 2015 and 2024 were included in the systematic review. The entire selection process followed the PRISMA 2020 guidelines (16) and is presented in the PRISMA flow diagram (Figure 1).

Inclusion criteria were formulated to ensure the relevance of the selected studies and to align with the aim of the systematic literature review. Only research sources meeting these predefined criteria were included:

1. Research examining the impact of the psychosocial work environment on nurses' health.
2. Studies assessing health using salutogenic indicators such as SHIS and WEMS.
3. Research articles published between 2015 and 2024.
4. Articles published in English or Lithuanian.

Exclusion criteria were designed to filter out irrelevant or inappropriate sources:

1. Research not related to nurses' health and the psychosocial work environment.
2. Studies that do not use salutogenic health assessment methods.
3. Articles published outside the 2015–2024 window.
4. Articles lacking relevant information or not meeting scientific quality requirements.

The search was conducted by entering keywords and combinations of keywords in the databases used, employing the logical operators “AND” and “OR” to filter for articles meeting the specified criteria.

Quality and risk-of-bias assessment were performed using appropriate critical appraisal checklists. The quality of the included articles was assessed with the Joanna Briggs Institute Critical Appraisal Tool (JBI) (17), the Mixed Methods Appraisal Tool (MMAT) (18) and, where applicable, the Critical Appraisal Skills Programme (CASP) (19), to ensure that only reliable and high-quality studies were included.

For each selected study, key elements related to study design, participant characteristics, aspects of the psychosocial work environment and nurse health assessment results were identified. This information was systematically categorized and summarized to identify recurrent trends and thematic links between different studies. Thematic synthesis was used for the analysis, enabling structured integration of qualitative and quantitative research data, identification of common patterns, and identification of existing research gaps in the field.

This methodological approach ensures the transparency and credibility, allowing for a systematic synthesis of the existing scientific information and provides sound conclusions and recommendations. The quality of the included studies was assessed using standardized tools (see Supplementary Table 1).

The articles selected for analysis systematically extracted information on study objectives, sample, assessment instruments used, and main results. The data were organized in a tabular format to facilitate comparison of study characteristics and to highlight common trends. An overview of the study characteristics and results is presented in Supplementary Table 2.

A large language model (LLM) was used to assist in drafting parts of the manuscript. The model was not used to generate primary results or to draw conclusions. Outputs from the model were reviewed and edited by the authors.

## 3 Results

The systematic review included 14 studies published between 2015 and 2024, encompassing diverse geographical contexts and methodological approaches. Most studies were conducted in Europe, with evidence from Sweden, Spain, Portugal, and Lithuania (2, 6, 11, 12, 20). Additional evidence came from Africa (Nigeria and Egypt) (21, 22), Asia (23), and South America (24). Several studies originated from Lithuania, including peer-reviewed articles and academic dissertations (25–28).

In terms of methodology, the included studies represented a mix of quantitative cross-sectional surveys (2, 21–23), and systematic reviews (6, 9, 24). Additionally, four Lithuanian dissertations and empirical works contributed national perspectives on nurses' psychosocial work environment and its relation to health outcomes (20, 25, 26, 28).

Sample sizes varied considerably, ranging from small hospital-based surveys with fewer than 100 participants (11) to large cross-sectional studies with several hundred respondents (2, 23). Systematic reviews (6, 9, 24) synthesized findings across multiple international contexts, strengthening the generalizability of results.

A variety of measurement instruments were used to assess psychosocial work environment and salutogenic outcomes. The Sense of Coherence (SOC) scale was frequently applied to capture nurses' resilience and stress resistance (9, 12). Other tools included the Salutogenic Health Indicator Scale (SHIS) and the Work Experience Measurement Scale (WEMS) (20), as well as broader instruments measuring job satisfaction, burnout, and engagement (2, 21, 22).

Positive psychosocial work environment factors were found to play a protective role in nurses' health and well-being. Social support from colleagues and supervisors consistently emerged as one of the strongest resources associated with higher job satisfaction, greater engagement, and reduced stress (2, 6, 11). Autonomy and job control were also critical, with several studies indicating that higher perceived control over work tasks correlated with lower burnout and better mental health outcomes (22, 23).

Conversely, negative factors such as high workload, time pressure, and workplace conflict were strongly associated with stress, emotional exhaustion, and reduced well-being. For example, Abdou et al. (21) highlighted that poor working conditions and lack of resources directly impaired performance and motivation among technical nurses, while Didbalyte et al. (25) and Jurgaitiene (26) reported that psychosocial stress significantly affected nurses' quality of life and perceptions of patient safety.

The SOC scale was central in several studies, revealing that higher SOC scores were linked to lower levels of stress, depression, and burnout among nurses (9, 12). SOC was also associated with better coping strategies and greater resilience, undescoring its importance as a salutogenic resource in challenging healthcare environments. Eriksson et al. (12) further contributed by developing and validating a new survey designed to measure sustainable working life for nurses, emphasizing the role of resistance resources against stress.

Studies using SHIS and WEMS provided complementary insights. Andruškiene et al. (20) found that SHIS scores revealed frequent reporting of physical and emotional symptoms linked to work stressors, while WEMS scores demonstrated strong associations between favorable work experiences and better health outcomes. These findings support the view that salutogenic indicators are practical tools for understanding the relationship between psychosocial work environment and health.

A wide range of health outcomes were reported across the studies. Mental health indicators such as burnout, depression, and stress symptoms were the most examined. Positive work engagement and job satisfaction were linked to supportive environments and higher SOC scores (2, 6). In contrast, excessive workload and lack of managerial support were repeatedly associated with emotional exhaustion, low motivation, and decreased quality of care (21, 25, 26).

Physical health outcomes, though less frequently measured, also demonstrated important associations. Nurses experiencing high psychosocial stress reported somatic symptoms such as fatigue, headaches, and sleep disturbances (25, 27). These results emphasize the interconnectedness of psychosocial environment and both mental and physical health dimensions.

The quality of the included studies was assessed using CASP, JBI, and MMAT checklists. Overall, most studies were rated as medium to high quality. Systematic reviews (6, 9, 24) demonstrated strong methodological rigor, while some dissertations and cross-sectional studies presented limitations such as small sample sizes, reliance on self-reported measures, or lack of longitudinal data (27, 28). Nevertheless, the overall evidence base was sufficient to identify consistent patterns linking psychosocial work environment factors and salutogenic indicators to nurses' health outcomes.

In summary, the review of 14 studies demonstrated that the psychosocial work environment plays a decisive role in shaping nurses' health and well-being. Positive factors such as social support, autonomy, and participatory leadership enhanced job satisfaction, engagement, and resilience, while negative factors including high workload, stress, and workplace conflict were associated with burnout, reduced motivation, and poorer health outcomes. Salutogenic indicators such as SOC, SHIS, and WEMS provided robust tools for understanding these dynamics, confirming their value in both research and practice. Taken together, the findings highlight the need for organizational interventions that strengthen salutogenic resources and reduce psychosocial risks to ensure a sustainable nursing workforce.

## 4 Discussion

The results of this systematic literature review confirm a close link between the psychosocial work environment and nurses' health. Across settings, favorable work environment factors, such as managerial support, colleague cooperation, clear job roles, and autonomy were associated with better well-being, whereas high workload, stress, conflicts, and insecurity were linked to poorer health indicators.

In Lithuania, Andruškiene et al. (20) reported that favorable working conditions corresponded with higher SHIS and improved WEMS ratings. Jurgaitiene (26) noted that emotional demands and insufficient managerial support were associated with a poorer work environment and weaker patient safety culture. Lithuanian master's theses by Rakutyte (27) and Šabonaite (28) further indicated that limited job control, job insecurity, and excessive demands relate to worse subjective employee health.

European studies beyond Lithuania show both convergence and nuance. In Sweden and other Nordic countries, Eriksson et al. (12) and Lindmark et al. (6) found that salutogenic resources, such as sense of coherence (SOC), and a supportive work environment, correlate with better employee health, motivation, and willingness to continue working. Systematic reviews (9, 24) indicate that higher SOC reduces burnout and depression and increases job satisfaction, while a supportive environment mitigates psychosocial risks. García-Iglesias et al. (2) reported a direct relation between psychosocial risks and work engagement with nurses' mental health. In Faria et al. (11), leadership and nurses' participation in decision-making were particularly important for a health-promoting work environment.

Notably, some studies indicate that the benefits of leadership support and decision-making involvement may depend on context (e.g., resource availability, staffing ratios) and can interact with workload and role clarity, suggesting that a one-size-fits-all approach may be insufficient.

In Africa, studies by Akinwale and George (22) and Abdou et al. (21) showed that social support, access to work tools, and collegial relationships contribute job satisfaction; these factors are especially salient in resource-constrained settings. However, some resources (e.g., tool availability) may buffer risks in some contexts but not others, pointing to context-specific threshholds for what constitutes a favorable psychosocial environment.

Across Asia, Zhang et al. (23) showed that the work environment, perceived personal health, and task structure significantly affect nurses' well-being, aligning with findings from other regions on the importance of working conditions and psychosocial resources.

Didbalyte et al. (25) found that organizational-level interventions to reduce psychosocial risks can improve the work environment and employee health, though empirical data in this area remain limited. Across these studies, inconsistencies emerge in how strength of organizational support translates into measurable health outcomes, with some settings showing robust associations for SOC and leadership participation, while others show weaker or context-dependent effects.

While the review confirms an association between the psychosocial work environment and nurses' health across settings, the variability between studies needs to be discussed in detail and critically assessed. Cultural contexts vary widely: from Lithuania, Sweden, Spain, and Portugal to African and Asian settings, potentially shaping perceptions of support, control, and job demands, as well as reporting of health outcomes. Measurement tools also differ (e.g., SHIS, WEMS, SOC), and reliance on self-report questionnaires raises concerns about social desirability bias and instrument equivalence, complicating data integration and cross-country generalization. Consequently, while patterns are broadly consistent, the generalizability of findings should be interpreted with caution, and future work should prioritize harmonized measures and longitudinal designs to better delineate causal pathways and contextual moderators.

## 5 Conclusions

This systematic review confirms that the psychosocial work environment decisively shapes nurses' health and well-being, as reflected by salutogenic indicators. Across international and Lithuanian studies, supportive organizational climates, characterized by autonomy, participatory leadership, and teamwork were linked to better health outcomes, higher job satisfaction, and lower burnout risk.

Persistent stressors such as excessive workload, limited opportunities for career development, and insufficient managerial support were associated with reduced well-being and higher turnover intention. Lithuanian nurses face specific challenges, including high work demands, emotional strain, and limited recognition, which undermine motivation and resilience.

Salutogenic instruments like the Sense of Coherence scale, Salutogenic Health Indicator Scale and Work Experience Measurement Scale proved useful for identifying not only risk factors but also protective resources that sustain health in demanding environments. They can can inform the interventions aimed at strengthening resilience and promoting sustainable working conditions.

Overall, the evidence undescores the value of organizational strategies that cultivate a positive psychosocial climate and enhance salutogenic resources. Addressing workload, leadership, and support structures is essential to improving nurses' health, maintaining professional commitment, and supporting the healthcare workforce's long-term sustainability.
