# Supplementary material for: Psychosocial hospital work environment and nurses' health assessed with salutogenic indicators: a systematic review
Source: Front Public Health. 2026 Jun 16;14:1738847. doi: 10.3389/fpubh.2026.1738847 (PMC13317071; doi:10.3389/fpubh.2026.1738847)
Supplement: Supplementary file 1 [file Data_Sheet_1.pdf]

Supplementary Material

Supplementary Table 1. Quality assessment of included studies

No.	Source	Assessment tool	Quality score	Weaknesses	Comments	Included/Excluded
1.	Abdou, Fatma F.; El Mola, Magda A.; Elewa, Amal H. Technical nurses' perception of work environment factors that affect their performance. Egyptian Nursing Journal 20(2): p 237-245, May-August 2023.	Checklist for JBI cross-sectional studies	5/7 (medium quality)	No ethical considerations provided; potential sources of bias not discussed	The study reveals the links between work environment and engagement but does not provide information on ethics and risk of bias.	Included
2.	Akinwale OE. George OJ. Work environment and job satisfaction among nurses in government tertiary hospitals in Nigeria. Rajagiri Management Journal, 2020; 14(1): 71-92.	NWI	6/7 (good quality)	Study context limited (only Nigerian government hospitals); possible cultural bias	The study clearly shows the impact of the work environment on job satisfaction but lacks deeper analysis of socio-cultural factors.	Included

3.	Andruškienė, J., Kuzmienė, A., Martinkėnas, A., Jurgutis, A., Ejlertsson, G., & Andersson, I. (2015). Psychosocial work experiences related to health: A study of Lithuanian hospital employees. *Work*, *53*(3), 669-677.	Checklist for JBI cross-sectional studies	6/8 (good quality)	No information on potential sources of bias provided	The study provides a detailed analysis of the impact of the psychosocial environment on health but does not sufficiently address the risk of selection and response bias.	Included
4.	Di Tecco, C., Nielsen, K., Ghelli, M., Ronchetti, M., Marzocchi, I., Persechino, B., & Iavicoli, S. (2020). Improving Working Conditions and Job Satisfaction in Healthcare: A Study Concept Design on a Participatory Organizational Level Intervention in Psychosocial Risks Management. International Journal of Environmental Research and Public Health, 17(10), 3677.	Not available	Not applicable	No empirical data available	This paper presents a prospective study focusing on organizational level interventions for psychosocial risk management in the health sector.	Excluded
5.	Didbalytė, M., Mikaliūkštienė, A., & Fatkulina, N. (2022). PSYCHOSOCIAL STRESS EXPERIENCED BY NURSES AND ITS IMPACT ON HEALTH AND QUALITY OF LIFE. *Health Sciences*, *32*, 190-195.	JBI Review Publication Evaluation List	6/8 (good quality)	Criteria for assessing included sources not specified, levels of evidence not distinguished	The study makes a good case for examining the impact of psychosocial stress on nurses' health but lacks a clear structure for analysing the sources.	Included

6.	Eriksson, M., Johannesson, E., Kerekes, N., Emilsson, M., Pennbrant, S., & Nunstedt, H. (2024). Development and Psychometric Test of the Salutogenic Survey on Sustainable Working Life for Nurses: Identifying Resistance Resources against Stress. *International Journal of Environmental Research and Public Health*, *21*(2), 198.	JBI tool for instrument development research/psychometric evaluation	7/7 (high quality)	No major weaknesses. The instrument is purposefully developed, empirically and theoretically sound.	The study has a robust methodology with the MEASURE method, reliability and validity assessment, suitable for the identification of nurses' health factors.	Included
7.	Faria, A., Carvalho, J., Ferreira, M. M., Vidal, D. G., & Teixeira, J. (2023). Salutogenic factors and hospital work environments: A cross-sectional study in a small Portuguese hospital. The Philippine Journal of Nursing, 91(1), 57-65.	JBI cross-sectional study checklist	6/8 (good quality)	Lack of detail on the selection method; no discussion of possible bias	The study looks in detail at the working environment for health promotion, but lacks a clear reflection on the validity of the selection and the wider applicability of the results	Included
8.	García-Iglesias JJ, Gómez-Salgado J, Ortega-Moreno M, Navarro-Abal Y. Relationship Between Work Engagement, Psychosocial Risks, and Mental Health Among Spanish Nurses: A Cross-Sectional Study. Front Public Health. 2021 Jan 26;8:627472.	JBI Cross-Sectional Study Checklist	8/8 (high quality)	Minor limitation - self-administered questionnaires may introduce response bias	The study reliably reveals the relationship between work engagement, psychosocial risk and mental health based on a large sample size and validated instruments.	Included

9.	Jurgaitienė, J. (2019). *Nurses' psychosocial work environment factors and attitudes towards patient safety* (Doctoral dissertation, Klaipėda University.).	JBI Cross-Sectional Research Checklist	6/8 (good quality)	No information on confidence intervals for statistics provided; in some cases precise derivative analyses of percentages or means are missing	The study meets most of the JBI criteria but lacks more detailed statistical data for deeper analysis.	Included
10.	Lindmark, T., Engström, M., & Trygged, S. (2023). Psychosocial Work Environment and Well-Being of Direct-Care Staff Under Different Nursing Home Ownership Types: A Systematic Review. *Journal of Applied Gerontology*, *42*(2), 347-359.	JBI checklist of systematic reviews	9/11 (good quality)	Lack of a detailed flowchart of the publication selection process; the methodological quality of some of the included studies was not discussed in detail	The study analysed 17 studies using the JBI tool, following PRISMA guidelines, and found that non-profit institutions are more likely to have better employee well-being.	Included
11.	Michele Masanotti, G., Paolucci, S., Abbafati, E., Serratore, C., & Caricato, M. (2020). Sense of coherence in nurses: a systematic review. *International journal of environmental research and public health*, *17*(6), 1861.	Checklist of JBI systematic reviews	9/11 (good quality)	Lack of a detailed flowchart of the publication selection process; methodological quality of some included studies was not discussed in detail	The study, following PRISMA guidelines and using the JBI tool, included 39 studies and showed that higher levels of SOC improve the health of nurses, reduce burnout and increase job satisfaction.	Included

12.	Pousa, P. C. P., & Lucca, S. R. D. (2021). Psychosocial factors in nursing work and occupational risks: a systematic review. *Revista brasileira de enfermagem*, *74*(suppl 3), e20200198.	Checklist of JBI systematic reviews	8/11 (good quality)	Lack of a detailed flowchart of the publication selection process; methodological quality of some included studies was not discussed in detail	The study, following PRISMA guidelines and using the COPSOQ questionnaire, revealed high job demands and their impact on nurses' well-being, highlighting the importance of managerial support and the need for stress reduction interventions.	Included
13.	Rakutytė, K. (2019). *Assessment of psychosocial work environment factors and subjective health of physicians working in X institution* (Master's thesis, Lithuanian University of Health Sciences (Lithuania)).	Checklist for the evaluation of JBI cross-sectional studies	6/8 (medium-high quality)	No explicit ethical considerations at the end of the publication; risk of selection bias not discussed	The study comprehensively assessed the relationship between psychosocial factors and subjective health in a large hospital sample using validated questionnaires and a clear methodology.	Included
14.	Šabonaitė, U. (2024). *Psychosocial work environment factors of health care professionals in relation to subjective health* (Master's thesis, Lithuanian University of Health Sciences (Lithuania)).	MMAT	4/5	No detailed information on the validation of the questionnaire is provided; lack of discussion on possible selection bias.	The study was well conducted, using a validated instrument and clear statistical methods, and the results directly reflect the purpose of the study.	Included

15.	Zhang, X., Zhang, C., Gou, J. *et al.* The influence of psychosocial work environment, personal perceived health and job crafting on nurses' well-being: a cross-sectional survey study. *BMC Nurs* **23**, 373 (2024).	CASP	8/10	1. The cross-sectional design of the study does not allow causality to be established. 2. The study sample includes only 7 ICUs in Beijing, so the results may be geographically limited.	The study provides important insights into the psychosocial work environment and its impact on nurses' well-being based on the JD-R model.	Included

Supplementary Table 2. Evidence summary of included studies

No.	Publication author, year	Purpose of the study	Sample	Methods	Description of the organisation	Main results	Statistical criteria	Significance level
1.	Abdou et al., 2023	To assess nurses' perceptions of work environment factors that affect their performance	(n = 96)	National Cancer Institute affiliated to Cairo University Hospitals	Work environment factors questionnaire (Parker et al., 2010; Oswald, 2012)	83.3% of the technical nurses reported that work environment factors have a significant impact on performance. 90.3% identified the availability of work tools and 87.7% identified collegial relationships. No statistically significant relationship was found with personal characteristics.	ANOVA	< 0.05

2.	Akinwale & George, 2020	To investigate the influence of work environment factors on job satisfaction of nurses in federal and state tertiary level hospitals in Lagos State	(n= 402)	Federal and state tertiary level hospitals in Lagos State e, Nigeria	Nursing Work Index (NWI) (Aiken & Patrician, 2000)	All seven factors (socio-political climate, administrative and managerial support, autonomy and responsibility, remuneration, supervision and working conditions, recognition and achievement, career development and promotion) are positively related to job satisfaction among nurses. Salary was the most important factor determining job satisfaction, followed by career development and promotion.	Hierarchical multiple regression	$p < 0.05$
3.	Andruški ene et al., 2015	To assess the salutogenic relationship between psychosocial work environment and health of Lithuanian hospital employees.	(n= 714)	Hospital	WEMS (Nilsson et al., 2013), SHIS (Bringsén et al., 2009)	Favourable working conditions, positive work experience and time commitment were most strongly associated with a high SHIS. Managerial position - higher WEMS; age 40-54 - lower WEMS. Doctors rated working conditions most favourably; nurses rated autonomy most favourably.	SPSS 19.0	$p<0.05$

4.	Didbalytė et al., (2022)	To analyse the scientific literature on psychosocial stress and its impact on health and quality of life among nurses.	None	None	Systematic search of PubMed and Google Scholar databases.	Nurses are exposed to many stressors in their professional activities. As a result of chronic stress, most nurses experience both physical and psychological health problems that affect their life satisfaction and overall quality of life.	Not specified	Not specified
5.	Eriksson, M. et al., (2024)	To develop and psychometrically evaluate a new questionnaire to identify general and specific sources of resilience that help nurses manage work-related stress.	(n= 475)	Hospital	Salutogenic Survey on Sustainable Working Life for Nurses (SalWork-N), developed based on the MEASURE approach and salutogenic health theory.	The questionnaire includes 21 statements divided into 7 domains: job satisfaction, professional role, work motivation, commitment, sense of belonging in the workplace, factors and conditions for staying in the profession, and workload.	CFI = 0.956, RMSEA = 0.065 (95% confidence interval: 0.059-0.072), chi-square = 508.752, degrees of freedom = 168	$p<0.001$
6.	Faria, A. et al, (2023).	To identify the working environment of nurses in a small	(n= 90)	Hospital	Nursing Practice Work Environment Scale (Ferreira & Amendoeira (2014))	The dimension with the highest score was "Leadership and management of the head nurse" (mean 3.3 out of 4,	SPSS 27; Cronbach's alpha = 0.92	Not specified

		Portuguese hospital, diagnose the situation and focus on a healthy working environment.				SD = 0.5). The lowest dimension was "Nurses' participation in hospital affairs" (mean 2.8, SD = 0.7). All areas had a mean above 2.5, which is considered a favourable working environment.		
7.	García-Iglesias JJ et al (2021).	Assessing work engagement, psychosocial risk and psychological well-being of Spanish nurses by analysing existing relationships, and their links to nurses' self-identified mental health problems.	(n= 1704)	Hospital and Primary health care centre	Copenhagen Psychosocial Questionnaire (CoPsoQ-istas21) (Moncada I Lluís S, Llorens Serrano C, Salas Nicás S, Moriña Soler D, Navarro Giné A. 2021); Utrecht Work Engagement Scale (UWES-9) (Wilmar B. Schaufeli and Arnold B. Bakker. 2003); General Health Questionnaire (GHQ-12) (David P. Goldberg 1972).	Emergency nurses reported higher CoPsoQ-istas21 and GHQ-12 scores, while primary care nurses reported higher UWES-9 scores. Factors relevant to mental health: self-perception of health and vigour at work.	Kruskal-Wallis	$P<0.05$

8.	Jurgaitienė J., (2019)	To analyse the psychosocial work environment factors and attitudes towards patient safety among nurses.	(n= 332)	Healthcare facilities providing multiprofile services	Copenhagen Psychosocial Questionnaire II (Kristensen et. al. (2004-2005)); Attitudes to Safety Questionnaire (J. B. Sexton et. al. (2006))	Psychosocial factors such as emotional demands, lack of managerial support, lack of fairness and lack of job evaluation have a negative impact on the working environment. Most of these factors act as sources of stress. Positive attitudes towards patient safety are associated with higher job satisfaction.	SPSS 17.0.1	$p<0.05$
9.	Lindmark, Engström, Trygged (2023)	To assess differences in psychosocial work environment and well-being between different types of nursing home ownership	17 studies involving 12 843 participants	For-profit and not-for-profit nursing homes	A systematic review using the Joanna Briggs Institute critical appraisal tools	Results were mixed, but tended to be more favourable for non-profit nursing homes, particularly in terms of staff exit intentions, organisational commitment and stress-related outcomes. Workload was higher in not-for-profit facilities, but this was offset by better labour resources.	Not specified	Not specified
10.	Masanotti, Paolucci, Abbafati, Serratore, Caricato (2020)	To assess the relationship between nurses' Sustainable Concept of Care (SOC)	27 studies	Various healthcare institutions	SOC scales (Antonovsky, 1987)	SOC was associated with lower levels of depression, burnout and job dissatisfaction; SOC may be a health enhancing resource	Not specified	Not specified

		and work environmen t and individual factors						
11.	Pousa & de Lucca (2021)	To identify key psychosocia l factors in nurses' work using the Copenhagen Psychosocia l Questionnai re	15 studies	Various healthcare institution s	Copenhagen Psychosocial Questionnaire (COPSOQ) (Tage S. Kristensen, Harald Hannerz, Annie Høgh, Vilhelm Borg. 2000)	High levels of cognitive and emotional demands and a fast pace of work are commonplace in nurses' daily lives. Management support has a positive impact on nurses' mental health. Physical and psychological violence and shift work have a negative impact on family life and increase fatigue.	Not specified	Not specified
12.	Rakutytė (2019)	To assess the psychosocia l factors of the work environmen t and their association with subjective health of doctors working in X institution.	(n= 230)	Personal health care institution s	Copenhagen Burnout Questionnaire (Kristensen et al. (2005));<br><br>Job Satisfaction Questionnaire (Spector, P.E. (1985));<br><br>SF-36 (Ware & Sherbourne (1992));<br><br>Copenhagen Psychosocial Questionnaire scales (Kristensen et al.,	41.3% of respondents reported needing specific skills at work; 48.7% experience moderate behavioural stress; 33% experience general burnout. Statistically significant relationships were found between stress and age, number of children, and length of service at (r = 0.162-0.172; p < 0.05). Longer years of work were associated with poorer physical and general	IMB SPSS 20.0	P<0.05

					NRCWE; COPSOQ III - Burr, H. et al. (2019))	health (r = -0.361 and -0.265; $p < 0.001$).		
13.	Šabonaitė (2024)	To analyse the association of psychosocial factors of the work environment with subjective health in health professionals	(n= 122)	Personal health care institutions	Job satisfaction questionnaire; Subjective health assessment questionnaire	Almost half of the respondents lacked job-specific skills, job control, and support from co-workers and supervisors. However, half felt low job demands and only a quarter felt high job insecurity. 49.5% of workers rated their health as good and 7.3% as poor. Better physical and mental health was due to greater influence and control over decision-making and lower job insecurity.	χ^2 test; Mann-Whitney U; Kruskal-Wallis tests	p<0.05
14.	Zhang et al, (2024)	To assess the impact of psychosocial work environment, perceptions of personal health and job design on nurses' well-being	(n= 655)	Teaching hospital	Job Skills Rating Scale (Tims M, Bakker AB, Derks D. 2012); Copenhagen Psychosocial Questionnaire (COPSOQ) (Yeh WY, Cheng Y, Chen CJ, Hu PY, Kristensen TS (2007)); Employee Well-being Scale subscale (Warr (1990))	Improved psychosocial work environment has a positive impact on nurses' health and job well-being; perceptions of personal health act as a mediator; job design acts as a moderator	SPSS 24.0	p<0,001
